# Demonstration of the Presence of the “Deleted” *MIR122* Gene in HepG2 Cells

**DOI:** 10.1371/journal.pone.0122471

**Published:** 2015-03-26

**Authors:** Ibrahim A. Y. Hamad, Yue Fei, Anastasia Z. Kalea, Dan Yin, Andrew J. P. Smith, Jutta Palmen, Steve E. Humphries, Philippa J. Talmud, Ann P. Walker

**Affiliations:** 1 Centre for Cardiovascular Genetics, BHF Laboratories, Institute of Cardiovascular Science, UCL, London, WC1E 6JF, United Kingdom; 2 Medical Biochemistry Department, Alazhar University, Damietta, 34511, Egypt; 3 Key Laboratory of Molecular Biophysics of the Ministry of Education, College of Life Science and Technology, Center for Human Genome Research, Huazhong University of Science and Technology (HUST), Wuhan, 430074, PR China; French National Center for Scientific Research - Institut de biologie moléculaire et cellulaire, FRANCE

## Abstract

MicroRNA 122 (miR-122) is highly expressed in the liver where it influences diverse biological processes and pathways, including hepatitis C virus replication and metabolism of iron and cholesterol. It is processed from a long non-coding primary transcript (~7.5 kb) and the gene has two evolutionarily-conserved regions containing the pri-mir-122 promoter and pre-mir-122 hairpin region. Several groups reported that the widely-used hepatocytic cell line HepG2 had deficient expression of miR-122, previously ascribed to deletion of the pre-mir-122 stem-loop region. We aimed to characterise this deletion by direct sequencing of 6078 bp containing the pri-mir-122 promoter and pre-mir-122 stem-loop region in HepG2 and Huh-7, a control hepatocytic cell line reported to express miR-122, supported by sequence analysis of cloned genomic DNA. In contrast to previous findings, the entire sequence was present in both cell lines. Ten SNPs were heterozygous in HepG2 indicating that DNA was present in two copies. Three validation isolates of HepG2 were sequenced, showing identical genotype to the original in two, whereas the third was different. Investigation of promoter chromatin status by FAIRE showed that Huh-7 cells had 6.2 ± 0.19- and 2.7 ± 0.01- fold more accessible chromatin at the proximal (HNF4α-binding) and distal DR1 transcription factor sites, compared to HepG2 cells (p=0.03 and 0.001, respectively). This was substantiated by ENCODE genome annotations, which showed a DNAse I hypersensitive site in the pri-mir-122 promoter in Huh-7 that was absent in HepG2 cells. While the origin of the reported deletion is unclear, cell lines should be obtained from a reputable source and used at low passage number to avoid discrepant results. Deficiency of miR-122 expression in HepG2 cells may be related to a relative deficiency of accessible promoter chromatin in HepG2 versus Huh-7 cells.

## Introduction

MicroRNA 122 (miR-122, encoded by EntrezGene ID 406906; OMIM *609582) is predominantly expressed in the liver [1]. MicroRNAs generally act by binding to partially complementary sequences in the 3’ untranslated region (UTR) of target mRNAs, resulting in either mRNA degradation or block of translation. However miR-122 may also act via a different mechanism, as it also binds to the 5’ UTR of the RNA genome of the hepatitis C virus (HCV), promoting viral replication [2]. MiR-122 participates in regulation of post-transcriptional gene expression in diverse physiological and pathological processes. These include support of the HCV viral cycle in hepatocytes, cell growth, apoptosis, carcinogenesis and regulation of hepatic iron and cholesterol metabolism [1–7]. Thus, antagonism of miR-122 decreased total plasma cholesterol levels whereas miR-122—mimics were shown to target the *HFE* and *HFE2* (*HJV*) genes, influencing hepcidin levels and thereby body iron absorption, in both animal models and man. A phase 2 clinical trial of miR-122 inhibition using the locked nucleic acid miR-122 inhibitor miravirsen for treatment of chronic HCV genotype 1 infection reported dose-dependent reductions in the level of HCV RNA. Serum total cholesterol levels were also decreased by ~25% over the 12 week period after miravirsen treatment [4–7]. Hence miR-122 may also be considered as a potential therapeutic target in non-alcoholic fatty liver disease, the metabolic syndrome and cardiovascular disease.

Reduced expression of miR-122 in hepatocellular carcinoma versus control liver tissue and implication of many of its target mRNAs in carcinogenesis suggested that miR-122 acts as a tumour suppressor in hepatocytes [8–10]. However, studies of the expression pattern of miR-122 in two models of human liver cancer reported that miR-122 is expressed in the Huh-7 cell line but deficient in HepG2 [3,9–13]. The potential basis of this difference in expression was investigated by cloning and sequencing the pre-mir-122 stem-loop genomic region, along with two sequences proposed at that time as putative promoter regions, from HepG2 and Huh-7 cells. The pre-mir-122 stem-loop region, but not the two upstream regions, was reported to be deleted only in HepG2 cells and this was suggested to be the cause of deficient miR-122 expression in these cells [9]. However, the results of the cloning experiment appeared inconclusive and the pri-mir-122 promoter was not investigated [9,14]. We therefore aimed to clarify the nature of any deletion of the *MIR122* gene in the genomic DNA of the widely-used HepG2 hepatocytic model cell line.

## Materials and Methods

HepG2 cells were obtained directly from European Collection of Cell Cultures (ECACC, Public Health England, Porton Down, England; catalogue no. 85011430, passage +101, vial date 12/04/11), where the identity of the cell line had been validated by short tandem repeat DNA profile and karyotype analyses. After 6 weeks storage in liquid nitrogen, the culture was established and after a single passage, the cell pellet was harvested for DNA preparation (HepG2-1). Validation isolate HepG2-2, from Dr Clare Selden, UCL (Royal Free Hospital Campus), UK was validated as HepG2 by analysis of both microsatellite genotypes and “DNA barcodes”; tested mycoplasma-free in three different assays; tested free of selected human pathogens and was shown to be sterile by direct inoculation analysis [15]. HepG2-3, from Professor Ann K. Daly, Newcastle University Medical School, UK, was originally obtained from ATCC. Details of the third laboratory isolate, HepG2-4, are available upon request. Control Huh-7 D12 cells (referred to as Huh-7; catalogue no. 01042712, passage 19) were obtained directly from the European Collection of Cell Cultures (Public Health England, Porton Down, England) [16,17]. HepG2 cells were cultured in Eagle's minimal essential medium with 10% foetal bovine serum (FBS) and 1% non-essential amino acids (Thermo Scientific SH3023801) and Huh-7 cells in Dulbecco's Modified Eagle Medium with 10% FBS and 2 mM glutamine under 5% CO_2_ at 37°C. Cultures were passaged at 70–80% confluence and seeded at 2–3 ×10^6^ cells per 75 cm^2^ flask.

Genomic DNA was extracted using the Nucleospin Blood QuickPure kit (Macherey-Nagel, Germany). Primers were designed using Primer3 (v. 0.4.0) [18,19] to amplify a 6078 bp region spanning the pri-mir-122 promoter and pre-mir-122 stem-loop region in 13 overlapping fragments for direct sequencing (Source Bioscience, Nottingham, UK; S1 Table) [9,14]. The sequence across a heterozygous, frame-shifting length polymorphism of a poly(T) tract was investigated by amplification of the miR122_735F / miR122_735R fragment, initially using *Taq* PCR Master Mix Kit (QIAGEN, UK). Fragments were cloned into pGEM-T Easy Vector System (Promega, UK) and transformed into *E*. *coli* XL-10 Gold (Stratagene / Agilent Technologies, UK) for plasmid purification using QIAprep Spin Miniprep kit (QIAGEN, UK) and sequencing using vector SP6 and T7 RNA polymerase promoter primers. Three HepG2 and five Huh-7 clones were sequenced. The cloning was repeated using Phusion high fidelity DNA polymerase (New England Biolabs Ltd, UK) and cloning the PCR products into Zero Blunt TOPO (Life Technologies Ltd, UK), transformation into *E*. *coli* TOP10 for plasmid purification using the QIAprep Spin Miniprep kit and sequencing using vector T3 and T7 RNA polymerase promoter primers of seven HepG2 and eight Huh-7 clones. The sequences were aligned to the GRCh37/hg19 (Feb. 2009) human genome assembly using ClustalW [20]. The Encyclopedia of DNA Elements (ENCODE) annotations and the positions of common SNPs were identified using the UCSC Genome Browser and dbSNP [21–23]. The 6078 bp region spanning the pri-mir-122 promoter and pre-mir-122 region was re-sequenced for validation in three additional HepG2 isolates from different sources. All primer sequences are listed in S1 Table.

Formaldehyde-Assisted Isolation of Regulatory Elements (FAIRE) was performed as previously described [24–26]. Sonication of genomic DNA from HepG2 and Huh-7 cells was performed using a UCD300 Bioruptor Next Generation sonication system (Diagenode s.a., Belgium) with a protocol of 20 cycles (1 min sonication, 1 min cooling; sonication level: high) at 4°C, in 100–300μl volumes (1.5 ml tubes), to obtain DNA fragments of 100–1000 bp, as determined by electrophoresis on 1% agarose, 0.5% Nusieve GTG agarose (Lonza) gels prepared in 8.9 mM Tris-borate buffer, 2mM EDTA pH 8.0 (1x TBE). Quantitative real-time PCR (qRT-PCR) analysis was performed in a 384 well plate using 10 ng FAIRE or non-FAIRE control DNA as template, 1x ABI Power SYBR Green mastermix #4367659 and 0.5 μM forward and reverse primers in 10 μl reactions. Primer pairs q122DR1-1F / q122DR1-1R and q122DR1-2F / q122DR1-2R (S1 Table) were designed for the proximal and distal DR-1 sites in the pri-mir-122 promoter, respectively [14]. Primers qHPRT1e3F / qHPRT1e3R were identified from ENCODE annotations to flank a well-defined, “gold standard” control site of inaccessible chromatin in HepG2 and Huh-7 cells under basal conditions, as recommended [27]. The qRT-PCR was performed using an ABI 7900HT Real-Time PCR System and the data were collected using RQ Manager v1.2 software. Three biological replicate cell culture experiments were analysed, each with four technical replicates. The comparative Ct method (2^-ΔΔCt^) was used to calculate the relative level of accessible chromatin, as the proportion of that in HepG2 [28]. No template controls were run in each assay to verify absence of PCR contamination. Data are presented as mean ± SEM and significance was determined using the Students’ paired, two tailed t-test.

## Results

**Fig 1:**
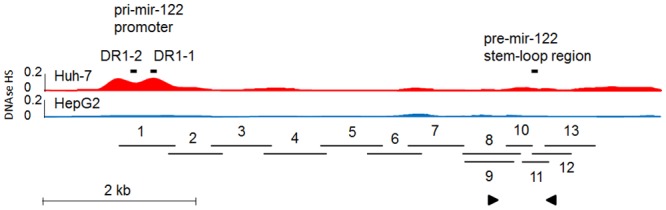
The “deleted” *MIR122* gene is present in two copies in HepG2 cells. A schematic diagram illustrates the human *MIR122* genomic locus. Li et al (2011) identified the pri-mir-122 promoter and the two DR-1 sites which were investigated by FAIRE in this study [14]. The position of the pre-mir-122 stem-loop region, previously reported to be deleted in HepG2 cells, is indicated. FAIRE showed that Huh-7 cells had 6.2 ± 0.19- and 2.7 ± 0.01- fold more accessible chromatin at the DR1-1 (proximal) and DR1-2 (distal) sites than HepG2 (p = 0.03 and 0.001), respectively. The ENCODE DNase I hypersensitivity (DNAse HS, relative units; Duke University) annotations of *MIR122* are shown as density signals for Huh-7 and HepG2 [22,23]. Consistent with the FAIRE results, these annotations showed a DNase I hypersensitivity site in the pri-mir-122 promoter in Huh-7 that was not seen in HepG2. The region was sequenced in 13 reactions (overlapping horizontal bars) and shown to be present in both Huh-7 and HepG2 cell lines (HepG2 isolates 1, 2 and 3). Ten SNPs spanning the region were heterozygous indicating that DNA was present in two copies. The positions of the miR-122 gene (upstream) and miR-122 gene (downstream) primers (Table 1) previously used to report the HepG2 *MIR122* “deletion” [9] are indicated by arrowheads; the downstream primer was located within an AluJb repeat sequence. The widely-reported deficiency of miR-122 expression in HepG2 cells is not caused by a *MIR122* deletion, but may be related to a less accessible chromatin conformation in HepG2 than Huh-7 cells. Scale bar, 2kb.

The pre-mir-122 stem-loop region, which was previously not detected in DNA from HepG2 cells and was reported likely to be mutated or deleted, was sequenced and shown to be present in the genomic DNA of HepG2 cells. The primer (miR-122 gene (downstream)) which Wu and colleagues previously used to report a deletion mapped within an Alu repeat sequence. This primer and its pair (miR-122 gene (upstream)) also contained one and three nucleotide differences, respectively, to the GRCh37 / hg19 (Feb. 2009) human genome assembly at their 5’ ends (Fig. 1) [9]. Direct sequencing of 6078 bp of genomic DNA containing the pri-mir-122 promoter and the pre-mir-122 hairpin region indicated identical sequence to chr18:56112983–56119060 of the GRCh37 / hg19 assembly, with the exception of 10 SNPs for which two different alleles were demonstrated in the original isolate HepG2-1, indicating that two copies of this genomic region were present in the genomic DNA of HepG2 cells (Fig. 1) [9,14,22]. Validation in three additional isolates of HepG2 cells showed identical genotype for the karyotyped- and “DNA barcoded”- reference isolate HepG2-2 and for HepG2-3, whereas HepG2-4 had a different genotype at all 10 SNPs (Table 1).

**Table 1:** Sequence validation of HepG2 identity confirmed the presence and heterozygosity of the pri-mir-122 promoter and pre-mir-122 stem-loop region.

**PCR**	**SNP**	**Genotype of cell line isolate**		
		**HepG2-1, HepG2-2 & HepG2-3**	**HepG2-4** ^a^	**Huh-7**
1	rs7227488	AG	AA	GG
1	rs60575556	GG	TT	GG
2	rs4245271	AG	AA	GG
4	rs4245272	AT	TT	AA
4	rs4940703	AG	AA	GG
4	rs4940704	GG	AA	GG
5	rs9319929	AC	AA	CC
6 & 7	rs9966765	CG	CC	GG
7	rs1135519	CT	CC	TT
11, 12 & 13	rs17669	CT	CC	TT
12 & 13	rs145725411	CT	CC	TT
13	rs6566969	AG	AA	GG

The pre-mir-122 stem-loop region, previously reported to be deleted in HepG2, was shown to be present in all isolates sequenced (PCRs 10, 11 and 12). Ten SNPs were heterozygous in three isolates of HepG2, indicating that DNA was present in two copies.

^a^The genotype of the isolate designated HepG2-4 indicated that it was probably not HepG2.

**Fig 2:**
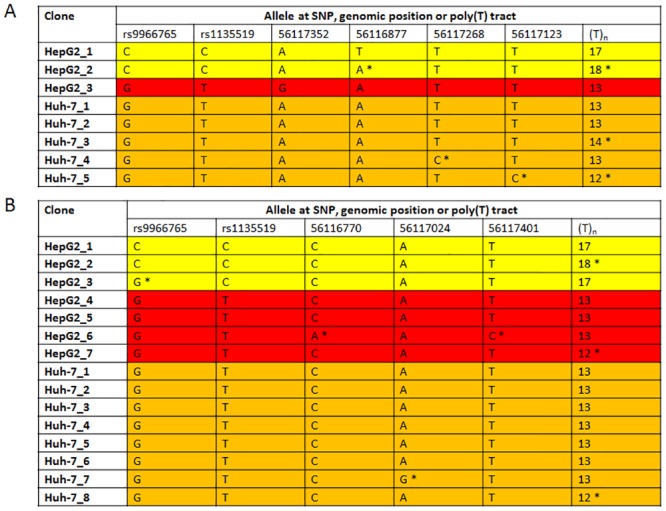
Cloned pre-mir-122 stem-loop region sequences from HepG2 DNA show two different haplotypes. (A) Cloned DNA sequences obtained after amplification with Taq polymerase. Two haplotypes (differently shaded) were observed for HepG2, consistent with the presence of two alleles across this region. However, among the eight HepG2 and Huh-7 clones, six sequence differences to the reference genome assembly were detected (*), so cloning was repeated using a proofreading DNA polymerase. (B) Cloned DNA sequences obtained after amplification with Phusion high fidelity DNA polymerase. Essentially the same two haplotypes of HepG2 were seen, but three novel single nucleotide substitution variants were detected and in a fourth clone, the rs9966765 allele did not correspond to the background haplotype observed. The reported error rate of Phusion High-Fidelity DNA Polymerase (GC Buffer) is 9.5 x 10^-7^ errors / base pair / PCR cycle (New England Biolabs). SNPs rs9966765 and rs1135519 are located upstream of the pre-mir-122 stem-loop region; their respective alleles are shown. The genomic positions on chromosome 18 (GRCh37/hg19 (Feb. 2009) human genome assembly) of non-SNP sequence variants and the alleles observed are shown; (T)_n_ refers to the length (base pairs) of the polymorphic poly(T) tract. *, position showing a sequence variant not corresponding to the predominant haplotypes observed.

The results from cloned single allele sequencing from the original HepG2-1 isolate confirmed that two of the upstream SNPs, rs9966765 and rs1135519, were heterozygous. In addition, analysis of the cloned sequences for a poly(T) length polymorphism located upstream of the pre-mir-122 stem-loop region (reported in dbSNP as rs143672020 and rs71173053) was consistent with the presence of two alleles for HepG2 (Fig. 2). The single allele cloned sequences obtained after amplification with Taq polymerase showed both an apparent slippage in the length of the poly(T) tract and sequence differences between clones at positions not corresponding to the positions of common SNPs (Fig. 2A). The cloning was therefore repeated after amplification using a proofreading DNA polymerase. Such changes were still observed after amplification with Phusion High Fidelity DNA Polymerase, with 3/15 (20%) of single allele clones obtained from HepG2 and Huh-7 DNA showing apparent poly(T) slippage and also four sequence variants observed that were not seen in other clones of the same haplotype (Fig. 2B). Overall, despite this sequence heterogeneity, the polymorphisms confirmed two different haplotypes in HepG2 DNA, consistent with the presence of two alleles of the pre-mir-122 stem-loop region.

Chromatin accessibility in the original HepG2-1 isolate was analysed by FAIRE at the two DR-1 (potentially HNF4α-binding) transcription factor sites described in the pri-mir-122 promoter [14]. Consistent with the reported deficiency of miR-122 expression in HepG2 cells, FAIRE showed that Huh-7 cells had 6.2 ± 0.19- and 2.7 ± 0.01- fold more accessible chromatin at the proximal and distal DR-1 promoter sites, (p = 0.03 and 0.001, respectively), than did HepG2 cells. Consistent with these findings, *in silico* analysis also indicated some regulatory differences between the two cell lines. A DNase I hypersensitive site was present at the pri-mir-122 promoter in Huh-7 cells that was absent in the HepG2 cells, indicating a difference in potential promoter activity and suggesting an effect on miR-122 expression (Fig. 1) [14,22,23].

## Discussion

MiR-122 is considered a potential therapeutic target as it regulates diverse processes and pathways, including HCV replication and hepatic iron and cholesterol metabolism. A clinical trial of miR-122 inhibition using miravirsen in the setting of HCV infection showed dose-dependent reduction of HCV RNA, along with a maximal reduction in serum total cholesterol concentration of ~ 0.8–1.7 mM and a sustained reduction 14 weeks after cessation of treatment of ~ 0.4–1.7 mM, depending on the dosage group [7]. The present study demonstrated that the region encompassing pri-mir-122 promoter and pre-mir-122 stem-loop was present in the HepG2 cell line, whereas deletion of the pre-mir-122 stem-loop region had been previously reported as the cause of low miR-122 expression in HepG2 cells [9,14]. This was supported by direct sequencing of PCR products and cloned DNA. Although “loss of heterozygosity” of tumour suppressor genes is a commonly observed step in tumourigenesis, there was no “loss of heterozygosity” of the *MIR122* gene in HepG2 cells: two different alleles were detected at 10 SNPs spanning the 6078 bp genomic region spanning the pri-mir-122 promoter and the pre-mir-122 stem-loop region [29]. The sequence of the original isolate, obtained and used directly from ECACC, was identical to two of three additional isolates of HepG2 obtained from different laboratories, one of which had been validated as HepG2 by karyotyping and “DNA barcoding”[15]. The third isolate also showed no evidence of deletion across the 6078 bp region sequenced; however, it had a different genotype at all 10 SNPs, indicating that this cell line was probably not HepG2. This observation emphasises the importance of obtaining cell lines from a reputable source and using them at low passage number. Overall, the data confirmed the presence of the region spanning the pri-mir-122 promoter and pre-mir-122 stem-loop region in the genome of HepG2 cells. Thus, deletion is not the cause of the widely reported low basal miR-122 expression in HepG2 cells [3,9–13]. This deficient expression could therefore result from chromatin or histone features either of the *MIR122* gene itself or of an enhancer or other remote regulatory sequence.

MiR-122 is predominantly expressed in the liver where it regulates several hepatic pathways and processes. The liver-enriched transcription factor HNF4α is an important regulator of miR-122 expression; in Huh-7 cells, HNF4α was previously shown to bind to the proximal conserved DR-1 site in the pri-mir-122 promoter [14]. The distal DR-1 site was not investigated. FAIRE was therefore used to investigate accessibility of chromatin at the two predicted DR-1 sites in the pri-mir-122 promoter. By FAIRE, Huh-7 cells had 6.2 ± 0.19- and 2.7 ± 0.01- fold more accessible chromatin at the proximal and distal DR1 sites, respectively, compared to HepG2 cells (p = 0.03 and 0.001, respectively). Consistent with these findings, *in silico* analysis of ENCODE annotations showed a DNAse I hypersensitive site in the pri-mir-122 promoter of the Huh-7 cell line that was absent from HepG2 cells. This indicated that under the basal conditions studied, in Huh-7 cells, DNA at this site was relatively accessible for binding of transcription factors whereas in HepG2, chromatin was condensed and inaccessible. Taken together, these data indicate that chromatin at two predicted HNF4α binding sites in the HepG2 promoter (one of which was previously demonstrated to bind HNF4α) is less accessible than that in Huh-7 cells, potentially leading to transcriptional repression. This mechanism could contribute to or explain the reported difference in miR-122 expression between the two cell lines.

Sequence heterogeneity of a poly(T) tract in the *MIR122* gene was detected by sequencing cloned DNA, which also supported the presence of two different alleles in HepG2 cells, although some clone haplotypes showed an apparent “slippage” in the length of the poly(T) tract and / or additional “non-SNP” single nucleotide substitutions in individual clones. The origin of this micro-heterogeneity of DNA sequence is not known, but it could have been present in the original tumour from which the HepG2 cell line was derived [16], or may have occurred during cloning or propagation of the cell line, or as a result of PCR or cloning. Similar sequence heterogeneity was observed for cloned Huh-7 DNA. The observed error rate was ~14-fold higher than that reported for Phusion High-Fidelity DNA Polymerase (GC Buffer) (New England Biolabs; Fig. 2) which, if this error rate is correct, suggests that these changes are unlikely to have arisen solely as a result of PCR errors. The spectral karyotype of HepG2 was previously reported to show widespread cytogenetic heterogeneity, with both aneuploidy and structural abnormalities of chromosomes (52–78, XY; del(1)(p22); +2; +der(6)t(6;17)(p10;q22); +14; +der(16)t(6;16)(q22;p13.3); +20 X 2; der(21)t(1;21)(p22;q10)). However, these cytogenetic changes did not involve chromosome 18, which contains the *MIR122* gene[30]. Other commonly used cell lines have been found to harbour gene deletions [31,32], underlining the value of confirming cell culture studies performed with easily manipulated cell lines in additional cell lines or in more physiologically-relevant systems such as primary cell cultures.

The original error leading to incorrect reporting of deletion of the pre-mir-122 stem-loop region [9] could have arisen due to failure of PCR amplification or cloning. The downstream primer used by these authors for PCR was within an Alu repeat, a family of highly repeated sequences that are estimated to be present in over a million copies in the human genome [33]. Hence, degeneracy of one PCR primer and primer-template mismatches could have contributed along with other factors, such as template quality, to failure of amplification of the pre-mir-122 stem-loop region from HepG2 genomic DNA. Alternatively, the isolate of HepG2 cells used by these authors could possibly have undergone homozygous deletion of *MIR122* if late passage number cells were used; or a failure of cloning could have led to the incorrect identification of an apparent deletion.

The HepG2 cell line has been widely used as model human hepatocytic cell line in many biological studies, with a PubMed search (HepG2; 02/03/15) showing 17,490 citations. HepG2 cells offer certain advantages over Huh-7 cells as a model system, such as the ability to polarise with tight junctions separating apical and basolateral poles, forming structures reminiscent of the bile canaliculi of intact liver. For example they have been proposed as a model in which to investigate the effects of hepatocyte polarity upon the HCV life cycle [34]. While HCV replication in the HepG2 cell line occurs at a level approximately 850 times lower than in Huh-7 cells, co-transfection of miR-122 and the HCV “receptor”, CD81, into HepG2 enables these cells to support the entire viral life cycle [35]. Therefore, the demonstration that the entire *MIR122* gene is present, and not deleted, is significant because it indicates the potential for induction of miR-122 expression in HepG2 cells by appropriate ligands, transcription factors and / or growth conditions.

In conclusion, contrary to the previously reported deletion, we found the entire locus spanning the pri-mir-122 promoter and pre-mir-122 stem-loop region is present in two copies in the widely used HepG2 cell line. Analysis of ENCODE DNAse I genome annotations and FAIRE investigation of HepG2 and Huh-7 cultures suggested that the reported lack of miR-122 expression in HepG2 cells may be related to repression of transcription by a relatively condensed chromatin structure within the pri-mir-122 promoter.

## Supporting Information

S1 Table Primers used for PCR, sequencing and qPCR. Bp, base pairs; Tm, the melting temperature used for PCR; n/a, not applicable. ^a^These previously reported PCR primers were found to be problematic and the downstream primer mapped within an AluJb repeat sequence.
(DOCX)
