# Supplementary material for: Demonstration of the Presence of the “Deleted” MIR122 Gene in HepG2 Cells
Source: PLoS One. 2015 Mar 26;10(3):e0122471. doi: 10.1371/journal.pone.0122471 (PMC4374784; doi:10.1371/journal.pone.0122471)
Supplement: S1 Table — Bp, base pairs; Tm, the melting temperature used for PCR; n/a, not applicable. aThese previously reported PCR primers were found to be problematic and the downstream primer mapped within an AluJb repeat sequence. (DOCX) [file pone.0122471.s001.docx]

# Supporting Information

**S1 Table. Primers used for PCR, sequencing and qPCR.** Bp, base pairs; Tm, the melting temperature used for PCR; n/a, not applicable. ^a^These previously reported PCR primers were found to be problematic and the downstream primer mapped within an AluJb repeat sequence.

| Primer (reference) | Sequence | PCR no. | Fragment size (bp) | Tm (°C) |
| --- | --- | --- | --- | --- |
| miR-122_P1F | GTTGGCGTGAACAAAGGAAT | 1 | 739 | 58 |
| miR-122_P1R | TGTTCAACACCTCCTGTTGC |  |  |  |
| miR-122_P2F2 | CTGAGTGATCAGGCCGTTCT | 2 | 636 | 59 |
| miR-122_P2R2 | TCATGCCTGTGTTTCTCACC |  |  |  |
| miR-122_P3F | GCCCCAACTTGGCTAAAAAT | 3 | 811 | 59 |
| miR-122_P3R | GCACTCGTCTTCCCAACAGT |  |  |  |
| miR-122_P4F | GGGCATTAAAAGAGGAGCAA | 4 | 829 | 59 |
| miR-122_P4R | CATTGCTAAAATGGCCAACA |  |  |  |
| miR-122_P5F | GAAAACCTTCTGCTTGTTTGTTT | 5 | 834 | 59 |
| miR-122_P5R | CACCCCAAATCAACCTCAAT |  |  |  |
| miR-122_P6F | TCTTGAATTTCCAAGTCTACCAGA | 6 | 701 | 61 |
| miR-122_P6R | TGTCCTTCCCTTTCCCTACA |  |  |  |
| miR-122_735F | CTGTTTCTTCCCAGCAGAGC | 7 | 735 | 55 |
| miR-122_735R | AGGGTTGGGAGCCAGTTATT |  |  |  |
| miR-122_740F | ACCCTTTCCCTTTTCAGCAT | 8 | 740 | 54 |
| miR-122_740R | TCTTGAGCAATGGGAAGAGC |  |  |  |
| miR-122_620 F | GCATTTGCAGGAAAAGAAGG | 9 | 620 | 54 |
| miR-122_620R | CAAACCCCTTCTGCAAAAAC |  |  |  |
| miR-122_425F | CATTCGGCCAGATTATTCTCA | 10 | 425 | 55 |
| miR-122_425R | CCATTGTCACACTCCACAGC |  |  |  |
| miR-122_357F | CCCGTGATGCTTCTTTTCTC | 11 | 357 | 55 |
| miR-122_357R | ATGTGAGAGGCAGGGTTCAG |  |  |  |
| miR-122_521F | GCTGTGGAGTGTGACAATGG | 12 | 521 | 61 |
| miR-122_521R | TCACTACAGCCTCAACCTCG |  |  |  |
| miR-122_651F | GTCTTGGCATCGTTTGCTTT | 13 | 651 | 55 |
| miR-122_651R | CTTGCGGTCTGTCTGCATAA |  |  |  |
| miR122 gene (upstream) | GCCCACAGGAAGTTTTTGCAGAAG | n/a | n/a^a^ | 57 |
| miR122 gene (downstream) | ATACTGCCCAGGCTGGACTTGAAC |  |  |  |
| T7 Promoter | TAATACGACTCACTATAGGG | n/a | n/a | n/a |
| T3 Promoter | ATTAACCCTCACTAAAG | n/a | n/a | n/a |
| SP6 promoter | ATTTAGGTGACACTATAG | n/a | n/a | n/a |
| q122DR1-1F | CTTGCTGAGTGTGTTTGACCA | n/a | 88 | 57 |
| q122DR1-1R | CGCATTATTTATCGATTCAGTGGG |  |  |  |
| q122DR1-2F | GGTCAAGAGCTCTACGTAGCA | n/a | 100 | 57 |
| q122DR1-2R | TGGTCACCTCCCCAGATTTC |  |  |  |
| qHPRT1e3F | ACTGAACGTCTTGCTCGAGA | n/a | 96 | 57 |
| qHPRT1e3R | GTCAGCAAAGAATTTATAGCCCC |  |  |  |
